# The Diagnostic Accuracy of Biological Markers of Sarcopenia, Sarcopenic Obesity, and Osteosarcopenia: Protocol for a Systematic Review and Meta‐Analysis

**DOI:** 10.1002/hsr2.71307

**Published:** 2025-10-07

**Authors:** Abigail N. Burton, Safoora Gharibzadeh, Mark P. Funnell, Kamlesh Khunti, Emma L. Watson, Thomas J. Wilkinson

**Affiliations:** ^1^ NIHR Applied Research Collaboration – East Midlands, Leicester General Hospital Leicester UK; ^2^ Leicester Real World Evidence Unit, Diabetes Research Centre, Leicester General Hospital University of Leicester Leicester UK; ^3^ Leicester Diabetes Centre University of Leicester Leicester UK; ^4^ Department of Cardiovascular Sciences University of Leicester Leicester UK

**Keywords:** biomarkers, diagnosis, meta‐analysis, sarcopenia, sensitivity and specificity, systematic review

## Abstract

**Background and Aims:**

Sarcopenia is the accelerated loss of skeletal muscle mass and function associated with aging, and can significantly impact a person's quality of life. Sarcopenia can also occur with other health conditions, such as excess adipose tissue and bone weakness. The presence of multiple long‐term conditions can exacerbate sarcopenia, and it has been observed in tandem with a variety of chronic illnesses. Biological markers can act as an early identifier or predictor of disease, enabling simple surveillance and prompt treatment. The need for effective screening and intervention has become more important as our population ages. This protocol outlines our approach to summarize the evidence on the diagnostic accuracy of various blood‐based biomarkers for diagnosing sarcopenia, including sarcopenic obesity and osteosarcopenia, through a systematic review and meta‐analysis.

**Methods:**

The protocol was developed in accordance with the Preferred Reporting Items for Systematic Reviews and Meta‐Analysis for Diagnostic Test Accuracy Studies (PRISMA‐DTA) 2018 statement and prospectively registered (PROSPERO: CRD42024609060). A comprehensive literature search using electronic databases including MEDLINE, Embase (via SCOPUS), CINAHL, Cochrane, and Web of Science, will be conducted. The review will include studies that meet the specific inclusion criteria, and the risk of bias in the included studies will be assessed using the Quality Assessment of Diagnostic Accuracy 2 quality assessment tool (QUADAS‐2). A meta‐analysis will be conducted using random‐effects approaches, based on the level of heterogeneity. Subgroup and sensitivity analysis will be performed if necessary.

**Results:**

The results of this review will be reported in accordance with PRISMA‐DTA standards, and published in a peer‐reviewed journal.

**Conclusion:**

The proposed systematic review and meta‐analysis will assess the diagnostic accuracies of potential blood‐based biomarkers for screening sarcopenia, sarcopenic obesity, and osteosarcopenia. The results will provide important evidence on the use of biomarkers to diagnose sarcopenia and its associated syndromes, which can inform the development of targeted screening and intervention in research and practice.

## 1 Introduction

Sarcopenia is a musculoskeletal condition resulting in progressive loss of muscle mass and function associated with aging, exacerbated by the presence of multiple long‐term conditions (MLTCs) [1]. Sarcopenia has been shown to result in poor physical performance, low quality of life, and greater hospitalization and mortality [2, 3, 4]. Additionally, it has been linked to other chronic conditions, such as diabetes mellitus and chronic obstructive pulmonary disease (COPD), in middle‐aged populations [5]. A recent review estimated a prevalence of 5%–13% in people between 60 and 70 years, 11%–50% of people over 80 years [6], and 31% in some chronic conditions (e.g., cardiovascular disease) [5].

Sarcopenia can occur in the presence of body composition‐related conditions such as obesity (sarcopenic obesity) or osteopenia/osteoporosis (osteosarcopenia). In sarcopenic obesity, adipose tissue expands and muscle mass is reduced, causing increased pro‐inflammatory cytokine levels and intramyocellular lipid deposits [7]. Populations with sarcopenic obesity are more closely associated with insulin resistance, metabolic syndrome, and have a higher cardiovascular disease risk than those with obesity or sarcopenia [8, 9]. Osteosarcopenia is defined as the presence of sarcopenia and osteopenia or osteoporosis, which are decreases in bone mineral density measured by T‐scores between −1 and −2.5 for osteopenia and osteoporosis, respectively [10]. When bone remodeling is impaired and formation does not keep pace with resorption, the imbalance in this mechanism leads to bone pathologies such as osteopenia, which then progresses to osteoporosis [11].

Sarcopenia assessment is commonly conducted through the objective measurement of muscle function and strength (e.g., handgrip strength, gait speed test) and muscle size (e.g., through advanced body composition imaging such as Dual‐Energy X‐Ray Absorptiometry [DEXA]) [12]. Nonetheless, the various and inconsistent diagnostic thresholds used to define sarcopenia and the short clinical time frames in which to screen patients can make objective and thorough assessment difficult, particularly in a clinical environment. Furthermore, many of the assessments required are often costly, resource‐intensive, potentially inappropriate for some individuals (e.g., radiation associated with DEXA), and require trained personnel to perform. Prompt, accurate, and easy diagnosis of sarcopenia for those with the condition and its associated syndromes is imperative as people are living longer and with greater MLTCs [13].

Biomarkers are indicators of biological processes that can be objectively measured, and provide the potential for an objective metric from which a diagnosis can be reached [14]. Early identification of at‐risk individuals enables disease to be monitored and preventative measures to be implemented, particularly for a progressive illness such as sarcopenia, where interventions have been shown to improve patient outcomes [15]. Identifying biomarkers for sarcopenia that can be monitored routinely (e.g., through blood tests) may help identify those at risk for early intervention and provide people with more healthy years of later life.

A previous systematic review by Lian et al. [16] evaluated the diagnostic accuracy of 30 blood‐based biomarkers of sarcopenia from 32 studies. The review found that the serum creatinine‐to‐cystatin C ratio had moderate diagnostic accuracy for sarcopenia, but the other biomarkers identified had low‐to‐moderate diagnostic accuracy. There were some limitations to the review; for example, several of the meta‐analyses were based on data from only two original studies. Additionally, when we attempted to replicate the searches, we found significantly more results than the number reported, suggesting that the search strategy may need refining and the searches updated. Given that this is a rapidly advancing area of research, and several known articles (including by our group) have been published since the Lian et al. review [17, 18, 19], an updated systematic review is justified.

The systematic review aims to evaluate the accuracy of various blood‐based biomarkers for identifying sarcopenia, sarcopenic obesity, and osteosarcopenia in any population. Our secondary outcomes will investigate any differences in accuracy between different demographics (e.g., sex, ethnicity) and clinical characteristics (e.g., MLTCs).

## 2 Methods

This systematic review and meta‐analysis protocol has been developed following the Preferred Reporting Items for a Systematic Review and Meta‐analysis of Diagnostic Test Accuracy Studies (PRISMA‐DTA) 2018 [20], and a PRISMA‐P checklist has been provided (Supporting Information: Material 3). The protocol for this systematic review has been prospectively registered in the International Prospective Register of Systematic Reviews (PROSPERO) (CRD42024609060).

### 2.1 Inclusion/Exclusion Criteria

The studies will be selected according to the criteria outlined in Table 1.

**Table 1:** Inclusion and exclusion criteria.

	Inclusion	Exclusion
Study design	Diagnostic test accuracy studies of any design.	Non‐peer‐reviewed work (such as theses, conference abstracts, etc.). Cross‐sectional studies if the analysis determining the accuracy of a biomarker against sarcopenia is performed using correlations or regression models.
Participants	Any age, gender, ethnicity, race, other socioeconomic and demographic characteristics, type of setting, or conditions.	
Exposures	Populations with sarcopenia, sarcopenic obesity, and osteosarcopenia. Populations with myopenia (muscle wasting) and dynapenia (loss of muscle strength), as these form key constructs of sarcopenia.	
Comparators	The studies must include a comparator group of populations without sarcopenia, sarcopenic obesity, or osteosarcopenia.	Studies without a control group.
Outcomes	Diagnostic test accuracy of a biomarker test in populations with and without sarcopenia, sarcopenia obesity, or osteosarcopenia. The primary outcome(s) will be the sensitivity (percentage of people with the condition who are correctly identified) and specificity (percentage of people without the condition excluded) of the biomarker. Secondary outcomes include other measures of accuracy (e.g., predictive values and likelihood ratios). A full list of outcomes can be found in Section 2.6.	
Setting	There will be no restriction by type of setting.	
Language	Studies written in English.	Studies written in languages other than English.

### 2.2 Information Sources and Search Strategy

PROSPERO will be searched for ongoing or recently completed systematic reviews, and the Cochrane Database of Systematic Reviews will be searched for similar completed reviews.

Literature searches will be developed using medical subject headings (MeSH) and test words relating to biomarkers, sarcopenia, osteosarcopenia, sarcopenic obesity, myopenia, dynapenia, sensitivity, and specificity. Terms related only to obesity will be excluded from the searches to limit the number of irrelevant results, and studies will only be included if they also contain any of the sarcopenic keywords. Literature searches will be conducted in MEDLINE, Embase (via SCOPUS), Cumulative Index to Nursing and Allied Health Literature (CINAHL), Cochrane, and Web of Science from database inception, following consultation with an experienced information specialist. Google Scholar (first 300 entries as per Haddaway et al. [21]) will also be searched. Database searches will be supplemented using forward and backward citation tracking from included studies. Due to variations in quality and lack of conventional peer review, we will not search for gray literature.

Supporting Information: Material 1 contains draft search strategies. Preliminary searches and scanning of results from MEDLINE, SCOPUS, and Web of Science returned several studies likely to be eligible upon full screening (Supporting Information: Material 2 – Seed Papers).

### 2.3 Selection Process

Literature search results will be uploaded to EndNote, where duplicates will be screened and removed. Results will then be transferred to review management software (Rayyan [22]), to facilitate collaboration between review team members. A calibration exercise will be performed to ensure team members understand the eligibility criteria.

Two review authors will independently screen titles and abstracts of search results against the inclusion criteria. We will obtain full reports for all articles that appear to meet the inclusion criteria, but reasons for title exclusion will not be recorded. One reviewer will screen the full‐text reports and decide whether these meet the inclusion criteria; a second review team member will verify these. We will seek additional information from study authors where necessary to resolve questions of eligibility, and disagreements will be resolved through discussion. We will record the reasons for excluding literature at this stage. Review authors will not be blinded to the journal titles, study authors, or institutions.

### 2.4 Data Collection

A data extraction form will be developed and piloted before full‐text data extraction. To reduce bias and errors in data extraction, data extraction per study will be completed independently by one reviewer with verification by another. To ensure consistency across reviewers, we will conduct calibration exercises before starting extraction, and reviewers will resolve disagreements through discussion. In the case of missing or unreported relevant data, two attempts will be made to contact the study authors to obtain the data.

### 2.5 Data Items

The extracted data will include but are not limited to author, year of publication, country of study, biomarker(s), participant characteristics (health conditions, age, sex, ethnicity/race), reference standard used, sample size, and outcomes (detailed below). Sociodemographic characteristics will be extracted using the Trial Forge PRO‐EDI tool [23].

### 2.6 Outcomes and Prioritization

#### 2.6.1 Primary Outcomes

The primary outcomes will be the sensitivity (percentage of people with the condition who are correctly identified) and specificity (percentage of people without the condition excluded) of the biomarkers.

#### 2.6.2 Secondary Outcomes

1. Positive predictive value;
2. Negative predictive value;
3. Positive likelihood ratio (PLR);
4. Negative likelihood ratio (NLR);
5. Area under the receiver operating characteristic curve (AUC‐ROC);
6. C‐index.

### 2.7 Quality and Risk of Bias Assessment

To assess the possible risk of bias for each study, two reviewers will use the Quality Assessment of Diagnostic Accuracy 2 quality assessment tool (QUADAS‐2) [24]. Disagreements will be resolved first by discussion and then by consultation with a third author for arbitration.

### 2.8 Data Synthesis

A systematic narrative synthesis of the information presented in the text and tables to summarize and explain the characteristics and findings of the included studies will be provided. The narrative synthesis will explore the relations and findings both within and between included studies, in line with guidance from the Center for Reviews and Dissemination.

If studies are sufficiently homogeneous regarding design and biomarker, we will conduct meta‐analyses using bivariate random‐effects models. These models will be based on sensitivity and specificity pairs and will be used to calculate pooled estimates of sensitivity, specificity, PLRs, NLRs, confidence intervals, and the AUC‐ROC. Skewed data and nonquantitative data will be presented descriptively.

Statistical heterogeneity will be tested using the chi‐squared test (*p*‐value significance level: 0.1) and *I* ^2^ statistic (significance level: *I* ^2^ ≥ 50%). If high levels among the studies exist (either *p* < 0.1 or *I* ^2^ ≥ 50%), the study design and its characteristics in the included study will be analyzed. We will try to explain the source of heterogeneity by subgroup or sensitivity analysis. Statistical analysis will be done using the STATA software, according to the statistical guidelines referenced in the current version of the Cochrane Handbook for Systematic Reviews of Diagnostic Test Accuracy. If statistical heterogeneity is observed, the hierarchical summary ROC model will be chosen. If heterogeneity is substantial, we will not perform a meta‐analysis; a narrative, qualitative summary will be done. Sensitivity analysis will be performed to explore the source of heterogeneity by omitting studies that are judged to be at high risk of bias. Subgroup analyses will be used to explore, where possible, sources of heterogeneity based on sex and ethnicity across different index conditions (e.g., diabetes, chronic kidney disease, etc.).

## 3 Discussion

The incidence of sarcopenia is expected to increase in line with the world's increasing population and aging population. The World Health Organization (WHO) expects that 1 in 6 people will be aged 60 years or over by 2030, and in 2050, 80% of older people will be living in low‐ and middle‐income countries [25]. This is especially important as sarcopenia is more prevalent in lower socioeconomic groups, as well as in those with lower education levels and the unemployed [26, 27]. Additionally, there are differences in the prevalence of sarcopenia between different ethnic groups [28, 29], with South Asian and Black African/Caribbean populations experiencing greater rates of early onset of MLTCs [30, 31].

The current management approach to sarcopenia involves the implementation of targeted nutrition and physical rehabilitation plans for patients [1]. Similar to the problems faced in diagnosing sarcopenia, the only way to monitor its progression is through imaging and strength testing. However, there are some patients for whom these methods are inappropriate (e.g., those with pacemakers and bedridden patients). Additionally, accessing full‐body scanners and ensuring the availability of specialist staff to operate and interpret results is much harder in rural communities and low‐income countries [32]. Alternatively, a simple blood test to monitor sarcopenia could be easily implemented through community‐based healthcare settings (e.g., mobile clinics, general practice) by less‐specialist staff. Such tests could then be implemented as part of routine care for older adults, with those flagged as potentially deteriorating referred for scans and further testing. Therefore, it is also possible that this review unearths a biomarker that is capable of being used as a monitor of condition severity, in addition to a diagnostic biomarker.

This systematic review with meta‐analyses will enable us to identify biomarkers of sarcopenia, sarcopenic obesity, and osteosarcopenia. It will compare the diagnostic accuracy of these markers, provide evidence on the most accurate in the current literature, and identify differences between population subgroups. Our results may help to support clinicians and researchers in the diagnosis and treatment of sarcopenia and its associated syndromes in at‐risk populations.

## Author Contributions

**Abigail N. Burton:** conceptualization, methodology, writing – original draft. **Safoora Gharibzadeh:** conceptualization, methodology, writing – review and editing, supervision. **Mark P. Funnell:** conceptualization, methodology, supervision, writing – review and editing. **Kamlesh Khunti:** conceptualization, methodology, writing – review and editing, supervision. **Emma L. Watson:** conceptualization, methodology, writing – review and editing. **Thomas J. Wilkinson:** conceptualization, methodology, writing – review and editing, supervision. All authors have read and approved the final version of the manuscript. All authors agree to be accountable for all aspects of the work.

## Conflicts of Interest

The authors declare no conflicts of interest.

## Transparency Statement

The lead author, Abigail N. Burton, affirms that this manuscript is an honest, accurate, and transparent account of the study being reported; that no important aspects of the study have been omitted; and that any discrepancies from the study as planned (and, if relevant, registered) have been explained.

## Supporting information

Protocol Manuscript Supplementary Material.

## Data Availability

The data that support the findings of this study are available from the corresponding author upon reasonable request.
