# Supplementary material for: The Diagnostic Accuracy of Biological Markers of Sarcopenia, Sarcopenic Obesity, and Osteosarcopenia: Protocol for a Systematic Review and Meta‐Analysis
Source: Health Sci Rep. 2025 Oct 7;8(10):e71307. doi: 10.1002/hsr2.71307 (PMC12504622; doi:10.1002/hsr2.71307)
Supplement: Supplementary file 1 — Protocol Manuscript Supplementary Material. [file HSR2-8-e71307-s001.docx]

**Supplementary Material 1 – Draft Search Strategies**

MEDLINE

1        exp Biomarkers/

2        (biomark* or biological mark* or marker* or factor* or indicator*).mp.

3        1 or 2

4        exp "Sensitivity and Specificity"/

5        exp Diagnostic Errors/

6        Likelihood Functions/

7        "Reproducibility of Results"/

8        exp Area Under Curve/

9        (accura* or false positive* or false negative* or false rate* or likelihood or probabilit*).mp.

10        ((observer adj variation*) or (predictive adj3 value*)).mp.

11        (receiver operat* or roc or sensitiv* or specific* or area under curve or auc).mp.

12        (c-index or c-statistic or concordance index or concordance statistic).mp.

13        4 or 5 or 6 or 7 or 8 or 9 or 10 or 11 or 12

14        exp Sarcopenia/

15        (sarcopeni* or myopeni* or dynapeni*).mp.

16        ((muscle or muscular or disuse) adj3 (atroph* or loss or wasting)).mp.

17        exp Muscular Atrophy/

18        osteosarcopeni*.mp.

19        14 or 15 or 16 or 17 or 18

20        3 and 13 and 19

Scopus (Embase)

TITLE-ABS-KEY("biomarker*" OR "biological mark*" OR "marker*" OR "indicator*" OR "factor*")

AND (

TITLE-ABS-KEY("sarcopeni*" OR "myopeni*" OR "dynapeni*")

OR TITLE-ABS-KEY("osteosarcopeni*")

OR TITLE-ABS-KEY("muscle" OR "muscular" OR "disuse" W/3 "atrophy" OR "loss" OR "wasting"))

AND (

TITLE-ABS-KEY("accurac*" OR "false positiv*" OR "false negativ*" OR "false rate*" OR "likelihood" OR "probabilit*" OR "receiver operat*" OR "ROC" OR "sensitiv*" OR "specific*" OR "area under curve" OR "AUC" OR "c-index" OR "c-statistic" OR "concordance index" OR "concordance statistic")

OR TITLE-ABS-KEY("observer" W/3 "variation*")

OR TITLE-ABS-KEY("predictive" W/3 "value*"))

CINAHL

S1 (MH "Biological Markers+") OR ( biomarker* or biological marker* ) OR ( indicator* or marker* or factor* )

S2 (MH "Reproducibility of Results") OR ( (MH "Sensitivity and Specificity+") ) OR (MH "Diagnostic Errors+") OR ( accura* or false positive* or false negative* or false rate* or likelihood or probabilit* ) OR ( receiver operat* or roc or sensitiv* or specific* or area under curve or auc ) OR ( c-index or c-statistic or concordance index or concordance statistic ) OR ( (observer N1 variation*) or (predictive N3 value*) )

S3 MH "Sarcopenia+" OR ( sarcopeni* or myopeni* or dynapeni* ) OR osteosarcopeni* OR ( (muscle or muscular or disuse) N3 (atroph* or loss or wasting) ) OR MH "Muscular Atrophy+"

S4 S1 AND S2 AND S3

Web of Science

1: biomarker* or biological marker* or marker* or indicator* or factor* (Topic)

2: sarcopeni* OR dynapenia* or myopeni* OR osteosarcopeni* OR (muscle or muscular or disuse) NEAR/3 (atroph* or loss* or wasting*) (Topic)

3: accura* or false positive* or false negative* or false rate* or likelihood or probabilit* (Topic) OR receiver operat* or roc or sensitiv* or specific* or area under curve or auc (Topic) OR c-index or c-statistic or concordance index or concordance statistic (Topic) OR (observer NEAR/1 variation*) or (predictive NEAR/3 value*) (Topic)

4: #3 AND #2 AND #1

**Supplementary Material 2 – Seed Papers**

Chen, R., et al., The triglyceride-glucose index as a novel marker associated with sarcopenia in non-diabetic patients on maintenance hemodialysis. Renal Failure, 2022. 44(1): p. 1616-1622.

Ladang, A., et al., Neurofilament-light chains (NF-L), a biomarker of neuronal damage, is increased in patients with severe sarcopenia: results of the SarcoPhAge study. Aging Clinical and Experimental Research, 2023. 35(10): p. 2029-2037.

Lo, C.-J., et al., Plasma acylcarnitine in elderly Taiwanese: as biomarkers of possible sarcopenia and sarcopenia. BMC Geriatrics, 2023. 23(1): p. 769.

Wilkinson, T.J., et al., Diagnostic accuracy of a ‘sarcopenia index’ based on serum biomarkers creatinine and cystatin C in 458,702 UK Biobank participants. Clinical Nutrition ESPEN, 2024. 63: p. 207-213.

Selçuk, N., et al., Sarcopenia is a risk factor for major adverse cardiac events after surgical revascularization for critical limb ischemia. Vascular, 2023. 31(1): p. 64-71.

**Supplementary Material 3 – PRISMA-P Checklist**

| **Section/topic** | **#** | **Checklist item** | **Information reported** | | **Page number(s)** |
| --- | --- | --- | --- | --- | --- |
|  |  |  | **Yes** | **No** |  |
| **ADMINISTRATIVE INFORMATION** | | | | | |
| **Title** | | | | | |
| Identification | 1a | Identify the report as a protocol of a systematic review |  |  | 1 |
| Update | 1b | If the protocol is for an update of a previous systematic review, identify as such |  |  |  |
| **Registration** | 2 | If registered, provide the name of the registry (e.g., PROSPERO) and registration number in the Abstract |  |  | 3 |
| **Authors** | | | | | |
| Contact | 3a | Provide name, institutional affiliation, and e-mail address of all protocol authors; provide physical mailing address of corresponding author |  |  | 2 |
| Contributions | 3b | Describe contributions of protocol authors and identify the guarantor of the review |  |  | 2 |
| **Amendments** | 4 | If the protocol represents an amendment of a previously completed or published protocol, identify as such and list changes; otherwise, state plan for documenting important protocol amendments |  |  | 5 |
| **Support** | | | | | |
| Sources | 5a | Indicate sources of financial or other support for the review |  |  | 2 |
| Sponsor | 5b | Provide name for the review funder and/or sponsor |  |  | 2 |
| Role of sponsor/funder | 5c | Describe roles of funder(s), sponsor(s), and/or institution(s), if any, in developing the protocol |  |  | 2 |
| **INTRODUCTION** | | | | | |
| **Rationale** | 6 | Describe the rationale for the review in the context of what is already known |  |  | 4 |
| **Objectives** | 7 | Provide an explicit statement of the question(s) the review will address with reference to participants, interventions, comparators, and outcomes (PICO) |  |  | 5 |
| **METHODS** | | | | | |
| **Eligibility criteria** | 8 | Specify the study characteristics (e.g., PICO, study design, setting, time frame) and report characteristics (e.g., years considered, language, publication status) to be used as criteria for eligibility for the review |  |  | 10 |
| **Information sources** | 9 | Describe all intended information sources (e.g., electronic databases, contact with study authors, trial registers, or other grey literature sources) with planned dates of coverage |  |  | 5 |
| **Search strategy** | 10 | Present draft of search strategy to be used for at least one electronic database, including planned limits, such that it could be repeated |  |  | SM Page 1 |
| ***STUDY RECORDS*** | | | | | |
| Data management | 11a | Describe the mechanism(s) that will be used to manage records and data throughout the review |  |  | 6 |
| Selection process | 11b | State the process that will be used for selecting studies (e.g., two independent reviewers) through each phase of the review (i.e., screening, eligibility, and inclusion in meta-analysis) |  |  | 5 |
| Data collection process | 11c | Describe planned method of extracting data from reports (e.g., piloting forms, done independently, in duplicate), any processes for obtaining and confirming data from investigators |  |  | 6 |
| **Data items** | 12 | List and define all variables for which data will be sought (e.g., PICO items, funding sources), any pre-planned data assumptions and simplifications |  |  | 6 |
| **Outcomes and prioritization** | 13 | List and define all outcomes for which data will be sought, including prioritization of main and additional outcomes, with rationale |  |  | 6 |
| **Risk of bias in individual studies** | 14 | Describe anticipated methods for assessing risk of bias of individual studies, including whether this will be done at the outcome or study level, or both; state how this information will be used in data synthesis |  |  | 6 |
| ***DATA*** | | | | | |
| **Synthesis** | 15a | Describe criteria under which study data will be quantitatively synthesized |  |  | 7 |
|  | 15b | If data are appropriate for quantitative synthesis, describe planned summary measures, methods of handling data, and methods of combining data from studies, including any planned exploration of consistency (e.g., *I* ^2^, Kendall’s tau) |  |  | 7 |
|  | 15c | Describe any proposed additional analyses (e.g., sensitivity or subgroup analyses, meta-regression) |  |  | 7 |
|  | 15d | If quantitative synthesis is not appropriate, describe the type of summary planned |  |  | 7 |
| **Meta-bias(es)** | 16 | Specify any planned assessment of meta-bias(es) (e.g., publication bias across studies, selective reporting within studies) |  |  |  |
| **Confidence in cumulative evidence** | 17 | Describe how the strength of the body of evidence will be assessed (e.g., GRADE) |  |  |  |
